# How DNA secondary structures drive replication fork instability

**DOI:** 10.1016/j.dnarep.2025.103913

**Published:** 2025-12

**Authors:** Aditya Sethi, María Fernández-Casañas, Billie Delpino, Gideon Coster

**Affiliations:** Genome Replication Lab, Division of Cell and Molecular Biology, Institute of Cancer Research, Chester Beatty Laboratories, London SW3 6JB, UK

**Keywords:** DNA replication, DNA secondary structures, Replication fork stalling, DNA accessory helicases, Replication stress, Genome instability, Single-stranded gaps (ssDNA gaps), Double-strand breaks (DSBs)

## Abstract

DNA secondary structures, such as hairpins, cruciforms, triplexes, G-quadruplexes and iMotifs, are common, dynamic features that replication forks routinely encounter. However, how these structures destabilise the replication fork remains unclear. Here, we propose a framework describing the immediate consequences of replication forks encountering DNA secondary structures. This review considers outcomes according to the affected strand (leading or lagging) and the timing of structure formation, linking strand geometry and folding dynamics to replisome behaviour. Stable, pre-formed structures on the leading strand template either impede, or are bypassed by, the CMG (CDC45-MCM-GINS) helicase, frequently leaving single-stranded DNA (ssDNA) gaps. Leading strand structures inhibit DNA polymerase ε (Pol ε), induce fork uncoupling, again producing post-replicative ssDNA gaps which can channel into fork reversal or PrimPol-dependent repriming. Lagging strand template structures inhibit DNA polymerase δ (Pol δ) and structures on 5′ flaps impair Okazaki fragment maturation (OFM); both impediments yield ssDNA nicks or gaps. In each case, replication protein A (RPA) availability and the replication checkpoint define a tolerance window and coordinate hand-offs to accessory helicases, Pol δ strand displacement synthesis, and translesion synthesis (TLS). Immediate double-strand breaks (DSBs) are unlikely as an immediate consequence. Instead, we propose strand-specific ssDNA gaps predominate and may later be converted into DSBs during late S/G2 processing, mitosis, or the next S phase. This review integrates mechanisms to connect structure dynamics with fork responses and downstream ssDNA gaps and breaks, providing possible models of structure-induced genome instability.

## 1 PART 1: normal replication fork progression and DNA secondary structures

### 1.1 Eukaryotic DNA replication

In the cell cycle, cells duplicate their entire genome during S phase so that at mitosis, each daughter cell inherits an identical chromosome set. Errors introduced at this stage range from single-base substitutions to large-scale rearrangements and can disrupt both coding and regulatory DNA. Replication-induced genomic instability is therefore a primary driver of genetic disease, most notably cancer [1]. Therefore, studying replication and its perturbation is key for potential therapeutic intervention.

**Fig. 1:**
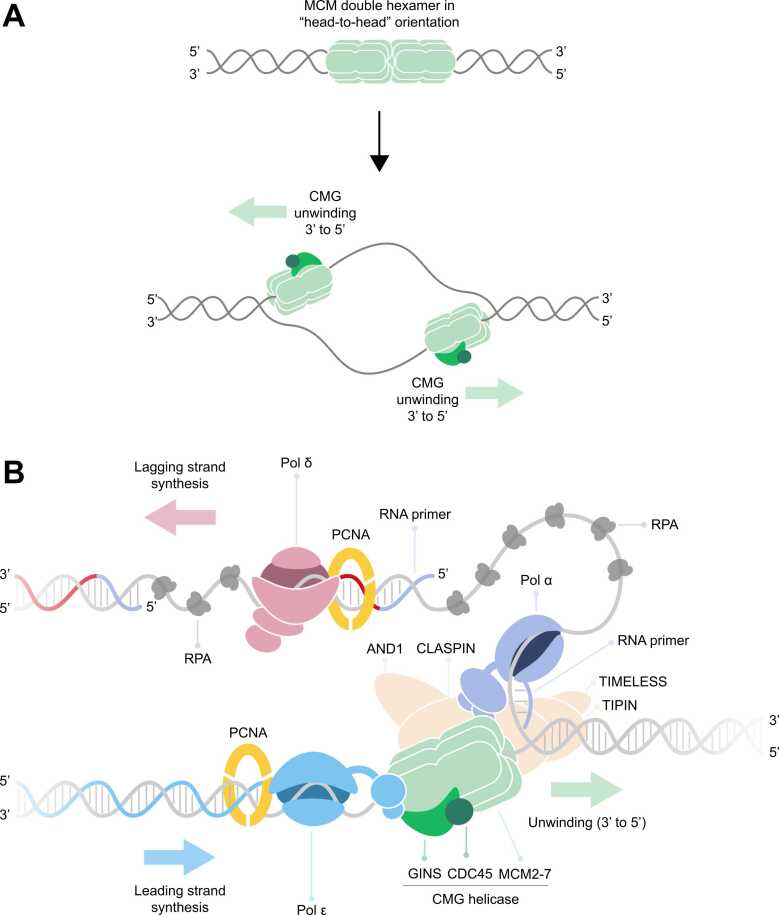
Eukaryotic replication fork progression. (A) Replication initiation: DNA replication initiates in two distinct steps. First, MCM double hexamers are loaded in a head-to-head orientation from the end of mitosis and throughout G1 phase. As cell enter S phase, activation produces two CMG helicases that translocate 3′→5′, establishing bidirectional forks. (B) Replication fork components: The CMG helicase translocates 3′→5′ on the leading strand template and unwinds parental DNA. The leading strand is synthesised continuously by Pol ε, which is tethered to CMG. The lagging strand is primed by Pol α and extended by Pol δ to form Okazaki fragments. PCNA clamps enhance polymerase processivity, RPA coats exposed ssDNA, and the fork protection complex (FPC) along with AND-1 coordinate unwinding with synthesis. Abbreviations: MCM, Minichromosome maintenance; CMG, CDC45-MCM-GINS; CDC45, Cell division control protein 45 homolog; GINS, DNA replication complex GINS protein; Pol ε, Polymerase epsilon; Pol δ, Polymerase delta; Pol α, Polymerase alpha; PCNA, proliferating cell nuclear antigen; RPA, replication protein A; FPC, fork protection complex; TIMELESS, Protein timeless homolog (Tof1 in yeast); TIPIN, TIMELESS-interacting protein (Csm3 in yeast); AND-1, replisome hub (Ctf4 in yeast); ssDNA, single-stranded DNA.

Preparations for DNA replication begin in G1 phase, when sites of replication initiation, termed replication origins, become competent for replication by loading of the core component of the replicative helicase, the MCM2–7 (Mini-chromosome maintenance) complex, in an inactive state. As cells transition into S phase, the loaded MCM is converted to its active form through the integration of CDC45 and the GINS complex to yield the CMG (CDC45-MCM-GINS) helicase “(CDC45: Cell division control protein 45 homolog; GINS: DNA replication complex GINS protein). These are highly regulated processes to ensure that each origin is activated (“fires”) only once per cell cycle. This ultimately leads to the simultaneous assembly of two replicative CMG helicases which unwind away from each other on opposite strands, thereby nucleating bidirectional fork progression (Fig. 1A) [reviewed in [2]]. During unwinding, CMG translocates in the 3′-5′ direction [3], [4], [5]. The steric exclusion model is widely accepted, whereby CMG translocates along the leading strand template while excluding the lagging strand template [5], [6]. Mechanistically, CMG translocation is driven by sequential ATP hydrolysis by different MCM subunits to pull single-stranded DNA (ssDNA) through its central channel to unwind parental double-stranded DNA (dsDNA) [7], [8] [reviewed in [9], [10], [11]]. After unwinding, each exposed strand serves as a template for DNA synthesis, which is carried out by three replicative DNA polymerases: Pol α (Polymerase alpha), Pol δ (Polymerase delta), and Pol ε (Polymerase epsilon) (Fig. 1B)*.* Pol ε synthesises the leading strand continuously in the 5’-3’ direction, as it is physically tethered to CMG, ensuring that minimal ssDNA becomes exposed [4], [12], [13]. Because of the anti-parallel nature of dsDNA, the lagging strand template runs in the opposite polarity and must therefore be synthesised backwards relative to the direction of CMG translocation. This occurs discontinuously as ∼200 nucleotide fragments called Okazaki fragments. The Pol α-primase complex repeatedly primes the lagging strand with a short RNA-DNA primer. This primer is extended by the main lagging strand polymerase, Pol δ, until it encounters and displaces part of the previous Okazaki fragment, generating a 5’ flap structure. This 5’ flap is processed by nucleases including FEN1 (Flap endonuclease 1), EXO1 (exonuclease 1) and DNA2 (DNA replication helicase/nuclease 2) [reviewed in [14]]. Subsequently, Okazaki fragments are ligated by LIG1 (DNA ligase 1), generating a continuous lagging strand. This process will be referred to as Okazaki fragment maturation (OFM).

The replisome also contains additional components required for efficient fork progression and coordination of related processes such as chromatin inheritance and DNA repair [13], [15]. RPA (Replication protein A) is a major eukaryotic ssDNA-binding protein which binds to exposed ssDNA generated due to the discontinuous nature of lagging strand synthesis, protecting it against damage and nucleolytic degradation [16]. PCNA (Proliferating Cell Nuclear Antigen) is a ring-shaped processivity factor that is loaded onto DNA by clamp loaders, RFC (Replication Factor C) and CTF18 (Chromosome transmission fidelity protein 18 homolog), enabling polymerases to incorporate more nucleotides per binding event. PCNA also serves as a landing pad for many factors, including OFM factors FEN1 and LIG1, as well as many DNA repair factors such as the Mismatch repair (MMR) machinery and Translesion Synthesis (TLS) polymerases [17]. Lastly, the Fork Protection Complex (FPC), comprised of TIMELESS-TIPIN (Tof1-Csm3 in yeast; TIMELESS: Protein timeless homolog; TIPIN: TIMELESS-interacting protein) and CLASPIN (Mrc1 in yeast), promotes efficient fork progression. AND-1 (Acidic nucleoplasmic DNA-binding protein) (Ctf4 in yeast) also plays roles in stimulating fork progression [18], [19] (Fig. 1B).

As DNA replication proceeds, the S phase checkpoint pathway constantly monitors its progress and coordinates it with DNA repair, the cell cycle, origin activation and control of the dNTP pool [20]. In the event of replication stress, the checkpoint triggers a global response. This is carried out by the ATM (Ataxia telangiectasia-mutated) and ATR (Ataxia telangiectasia and Rad3-related protein) kinases in mammalian cells (Tel1 and Mec1 in yeast). ATM is activated by double-strand breaks (DSBs) and triggers CHK2 (Checkpoint kinase 2), while ATR is activated by ssDNA and stalled forks, triggering CHK1 (Checkpoint kinase 1) [21], [22]. These kinases execute the S phase checkpoint via phosphorylation of a range of targets, including replisome factors, to promote damage repair before the cell cycle proceeds. A similar checkpoint exists to protect the transition from G2 to mitosis, also involving the ATM/ATR kinases [reviewed in: [23], [24], [25]].

In addition to copying genetic information, cells must also maintain epigenetic information. Therefore, the replication machinery also contains multiple factors and activities that facilitate the inheritance of parental epigenetic marks and histones onto daughter DNA. This process is termed replication-coupled nucleosome assembly (RCNA) [reviewed in [26], [27]]. To maintain nucleosome density and chromatin state after replication, both parental and newly synthesised histones are deposited on the nascent strands. This process is tightly regulated and involves various histone chaperones, including components of the replisome such as FACT (Facilitates Chromatin Transcription), CAF-1 (Chromatin assembly factor 1), MCM2, Pol α, Pol ε and CLASPIN; perturbing these interactions can compromise (epi)genome stability, by disrupting nucleosome assembly and faithful propagation of histone marks, leading to aberrant chromatin states and accumulation of DNA damage [28], [29], [30], [31].

Finally, replication terminates when two converging forks encounter each other, leading to unloading of CMG from DNA via polyubiquitylation of MCM7. This is followed by decatenation, whereby interlinked DNA molecules that were formed due to topological entanglement are separated. Interestingly, PIF1 (ATP-dependent DNA helicase PIF1/Petite Integration Factor 1), an accessory helicase known to unwind G-quadruplexes (G4s) (see Section 1.2), has been shown to also promote completion of DNA synthesis and replication termination in *Saccharomyces cerevisiae* [32] [reviewed in [33], [34]].

Smooth progression of the replication fork relies on two key activities: the unwinding of dsDNA by CMG, and the synthesis of nascent DNA by polymerases. These activities can be affected by various obstacles that impede their smooth progression, including DNA base lesions, covalently and non-covalently bound proteins, and transcription-replication conflicts (TRCs) [reviewed in [35], [36], [37], [38], [39]]. Besides these obstacles, DNA secondary structures can also act as obstacles to DNA replication, which will be the focus of this review.

### 1.2 DNA secondary structures

In cells, DNA predominantly exists as the canonical right-handed double-helix formed by Watson-Crick base pairing, called B-DNA. However, DNA can also adopt alternative non-B DNA structures, referred to as DNA secondary structures. Roughly 10–15 % of the human genome is thermodynamically capable of folding into non-B conformations [40], [41]. DNA secondary structures, such as hairpins, cruciforms, triplexes, G4s and intercalated motifs (iMotifs), are dynamic elements that contribute to physiological functions such as transcription, replication timing, chromatin topology and telomere maintenance [secondary DNA structures reviewed in [41], [42], [43], [44], [45], [46]].

However, DNA secondary structures can also act as obstacles to the replisome and disrupt genome integrity. Unresolved DNA secondary structures can induce mutations, repeat expansions, copy-number changes and chromosomal rearrangements that contribute to a range of human diseases, including cancer and neurodegenerative repeat-expansion disorders [reviewed in [41], [42], [43], [44], [45], [47], [48]]. Therefore, understanding how genomic instability is induced by DNA secondary structures is of crucial importance. In this review, we focus on the immediate consequences of replication forks encountering secondary structures. Much of our understanding of this topic has been derived from studies utilising chemical stabilisation of secondary structures; while these studies can be informative, they can also bias structure formation and skew data interpretation accordingly (see Box 1 for more detail).

Box 1 **Chemical stabilisation of DNA secondary structures.**

Chemical stabilisation of DNA secondary structures is a standard experimental strategy for probing their biology. Benzoquinoquinoxaline (BQQ) derivatives selectively stabilise Y·R-Y triplex DNA and have been used to study triplex DNA biology [188], [189]. A coralyne-based fluorescence intercalator-displacement assay now permits rapid screening of additional triplex stabilisers [190]. G4 ligands have been the most extensively studied: pyridostatin (PDS), telomestatin (TMS), Phen-DC3, TASQ, BRACO-19 and CX-5461 are widely used cellular probes for G4s that exposed their roles in transcription, replication and telomere stability [191], [192], [193], [194], [195], [196], [197], [198], [199]. G4 ligands have been functionalised for a wide range of applications: for example, fluorogenic ligands enable live-cell imaging [110], [200], [201], [202], [203]. Comprehensive overviews of G4 ligand chemistry as well as translational perspectives can be found in [113], [204], [205], [206], [207], [208], [209]. The repertoire of validated iMotif ligands remains limited. Mitoxantrone stabilises promoter and telomeric iMotifs at physiological pH in vitro, although direct in-cell stabilisation has not been firmly established. At the BCL2 (B-cell lymphoma 2) promoter, the C-rich strand exists in a dynamic iMotif and hairpin equilibrium: IMC-48 biases toward the iMotif (upregulating BCL2), whereas IMC-76 biases toward the hairpin (downregulating BCL2) [158], [159]. However, later work indicated weak iMotif binding for IMC-48 [210]. Consistent with complementary-strand antagonism, some ligands stabilise G4s while destabilising iMotifs, reinforcing that readouts reflect on-target structure modulation plus chemical-specific pharmacology [112], [211], [212], [213]. The discovery and characterisation of iMotif ligands are compiled in current reviews [147], [148], [214], [215].

Chemical stabilisers, particularly G4 ligands, also have therapeutic applications [reviewed in [207], [216], [217]]. These ligands are invaluable chemical tools because they stabilise otherwise transient DNA secondary structures in cells and make them experimentally tractable. However, this can also bias detection and interpretation. Many ligands favour particular topologies (e.g., parallel G4s) and show incomplete G4-over-dsDNA selectivity, thereby reshaping which sites appear “on-target” [217], [218], [219]. Ligands can also cause off-target effects, notably CX-5461, which acts as TOP2 poisons, introducing ligand-specific routes to DNA damage [220], [221]. Finally, “ligand-as-reporter” strategies (e.g., BrdU-tagged probes) remain binding proxies rather than direct structure maps and thus require orthogonal validation [222]. Accordingly, genome instability readouts obtained under ligand treatment should be interpreted with caution, as they may reflect ligand pharmacology (e.g., topoisomerase trapping, R-loop amplification) in addition to genuine effects on DNA secondary structures.

#### 1.2.1 Hairpins and cruciforms

**Fig. 2:**
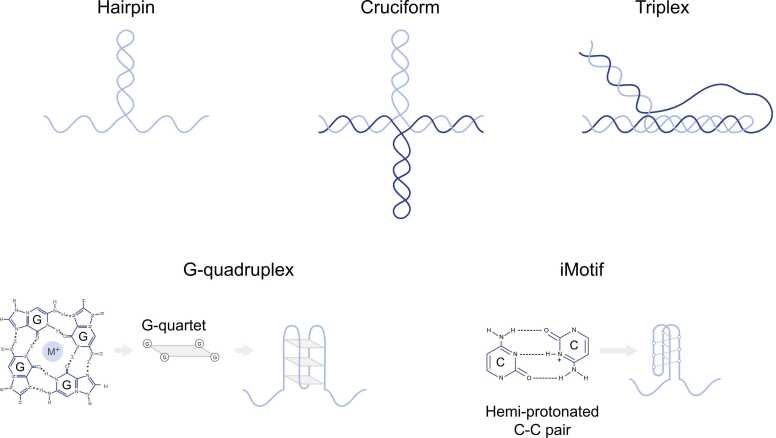
DNA secondary structures. Schematic representation of relevant DNA secondary structures, including hairpin and cruciform (extrusion of inverted repeats), triplex DNA (third strand bound in the major groove via Hoogsteen or reverse-Hoogsteen pairing), G-quadruplex (G4) (stacked G-quartets stabilised by a monovalent cation), and iMotif/iM (intercalated duplex stabilised by hemi-protonated C·C⁺ pairs). Abbreviations: G, Guanine; C, Cytosine.

Hairpins and cruciforms arise when inverted-repeat sequences fold onto themselves via canonical Watson-Crick base pairing. They either extrude under negative supercoiling [49], [50] or form readily in single-stranded contexts, such as exposed ssDNA on the lagging strand template [51], [52], [53], [54]. A single-strand extrusion produces an intra-strand hairpin, whereas extrusion of both strands generates a four-way cruciform resembling a Holliday junction (HJ) (Fig. 2) [55], [56], [57]. Genome-wide analysis shows that cruciform-forming repeats are enriched at promoters, replication origins, transcription terminators and centromeres in bacteria and eukaryotes [58], [59], [60], [61], [62]. Functionally, these motifs modulate transcription, origin activity and centromeric chromatin, yet long or closely spaced AT-rich repeats drive deletions and translocations in yeast and mammals [51], [63], [64], [65]. Importantly, repeat expansions that are characteristic of Huntington’s disease and myotonic dystrophy are fuelled by imperfect hairpin (or hairpin-like) intermediates formed during the replication of (CAG)/(CTG) repeats [66], [67], [68]. Furthermore, inverted repeats and hairpin loops are shown to drive DSBs, rearrangements, fragility and repeat-length changes [69], [70], [71] [hairpins and cruciforms reviewed in [47], [72]].

#### 1.2.2 Triplex DNA (H-DNA)

Triplex DNA forms when a homopurine-homopyrimidine mirror repeat (≥ 30 nt) within duplex DNA is locally unwound under negative supercoiling, and one of the single strands fold back onto the remaining duplex, binding in the major groove via Hoogsteen (Y·R-Y) or reverse-Hoogsteen (R·R-Y) hydrogen bonds [73] (Fig. 2). Pyrimidine triplexes (Y·R-Y), where the third strand is composed of pyrimidines, are favoured by mildly acidic pH whereas purine triplexes (R·R-Y) are stable at physiological pH [74], [75]. Triplex-forming sequences cluster in developmental gene introns, at chromatin loop anchors, and in antibody switch regions and can be visualised in vivo [76], [77], [78]. Importantly, experimental mapping of triplexes via S1-END-seq shows thousands of triplex sites that peak in S phase, indicating a tight relationship between structure folding and replication [79]. Triplex-forming repeats can promote mutations and chromosomal rearrangements, as these sequences are mutagenic, predispose to repeat expansions and chromosomal fragility, and are recurrently expanded in some cancers [80], [81], [82]. Long (GAA)ₙ tracts (>65 repeats) in the first intron of the FXN (Frataxin) gene adopt triplex structures, which cause the massive expansions that drive the pathology of Friedreich’s ataxia [83], [84], [85], [86] [triplex DNA reviewed in [87], [88]].

#### 1.2.3 G-quadruplexes (G4s)

G4s arise when four guanines form Hoogsteen hydrogen bonds to form a planar G-quartet and successive quartets stack around a central monovalent cation [89] (Fig. 2). G4s can exist as unimolecular, bimolecular or tetramolecular assemblies that adopt parallel, antiparallel or hybrid topologies [90], [91], [92]. In parallel G4s all contributing strands are oriented in the same 5’-3’ direction, whereas antiparallel structures have strands oriented in opposing directions. The architecture of G4s is dictated by G-tract lengths, loop size, loop base composition and bound cation [93], [94], [95], [96]. In silico analysis and high-throughput biochemical assays such as G4-seq predict that the human genome contains ∼700,000 G4-forming motifs [97], [98], [99], [100], [101], [102], [103], [104]. The development of an antibody that binds to G4s, called BG4, enabled researchers to show that only a subset of these sites folds into G4s at any given moment [104], [105], [106], [107], [108], [109]. Many studies also revealed the highly dynamic nature of G4 folding in cells, where hundreds of G4s rapidly form from early- to mid-S phase [107], [110], [111], [112]. However, these results represent a very small fraction of the estimated potential G4-forming sequences throughout the genome. The different methods of G4 mapping have been reviewed systematically in [113].

Functionally, G4s play important roles in gene expression, 3D genome organisation, telomere regulation and immunoglobulin class-switch recombination [104], [114], [115], [116], [117], [118], [119] [G4s reviewed in [120], [121], [122]]. However, when G4s persist or are not efficiently resolved, they can be deleterious. At regulatory regions, unresolved G4s interfere with normal chromatin reassembly and repair, creating heritable open chromatin states that are repeatedly exposed to replication stress and error-prone repair, and are therefore associated with elevated local substitution and indel rates [123], [124], [125], [126], [127]. Engineered G4s stall replication and require PIF1 helicase to prevent breakage in yeast [128], [129], [130]. A single persistent G4 can induce genome rearrangements that arise in one generation and are stably transmitted over multiple subsequent generations [131]. G4 stabilisation further promotes micronuclei formation [132]. G4-induced replication stress and genome instability are particularly evident in certain genetic backgrounds, such as ATRX-deficient malignant glioma, where G4 stabilisation drives DNA damage and reveals a targetable vulnerability [133]. More broadly, G4s have been linked to increased breakpoints and chromosomal rearrangements in cancer and are associated with common fragile sites (CFS) and oncogene promoters [134], [135], [136], [137], [138], [139], [140], [141].

#### 1.2.4 Intercalated Motif (iMotif/iM)

iMotifs are four-stranded structures that form when four tracts of cytosines assemble into two intercalated antiparallel duplexes stabilised by hemi-protonated C·C⁺ pairs [142], [143] (Fig. 2). Despite early scepticism about their physiological occurrence due to their requirement for acidic pH, iMotifs have since been shown to transiently form under physiological conditions [144], [145], [146] [iMotifs reviewed in [147], [148], [149], [150]].

The current consensus motif for a physiological iMotif is C_5_(N_1–19_ C_5_)_3_ [151], [152]. Since this motif requires longer C-tracts relative to the shorter G-tracts required for G4s, the frequency of iMotifs is expected to be much lower than G4s. Consistent with this, in silico analyses predict ∼ 5000 iMotif-forming sequences in the human genome capable of folding at neutral pH [150], [151], ∼ 140-fold fewer than the number of canonical G4s. Moreover, G4-iM Grinder likewise reports putative iMotif sequences to be markedly less abundant than G4-forming sequences [153]. This also means that while most G4-forming sequences will not harbour an iMotif-forming sequence on the complementary strand, most iMotif-forming sequences will harbour complementary G4-forming sequences [147], [150], [154]. In line with this interplay, biophysical and cellular studies indicate that G4 and iMotif structures generally form in a mutually exclusive, competitive manner at a given locus [112], [150], [155]. In vivo studies of iMotif formation utilise the iMotif-specific antibody iMab [112], [145], [146], [156] or the recently developed fluorescent probe IMCC-6 [157]. iMotifs are enriched at promoters and telomeres and are also detected at centromeric α-satellite DNA, where they can modulate transcription, telomerase activity and CENP-B loading, with implications for kinetochore assembly [145], [158], [159], [160], [161], [162], [163], [164]. Although the evidence is limited, iMotifs may contribute to local genomic instability, as iMotif sequences that are likely to exist at neutral pH in human cells correlate with spontaneous deletions [165].

#### 1.2.5 R-loops and G-loops

R-loops are three-stranded RNA-DNA hybrids, where RNA hybridises to its complementary DNA strand and displaces the other DNA strand. R-loops were first described as crucial intermediates for replication initiation of the ColE1 plasmid [166]. Since then, they have been shown to form naturally during transcription, DNA replication, and DNA repair and are also found at telomeres and centromeres [reviewed in [167]]. R-loops are relevant in the context of Transcription-Replication Conflicts (TRCs), which occur when the transcription and replication machineries collide with RNA Polymerase II, R-loops and/or supercoiling acting as replication obstacles [38], [168]. Importantly, TRCs are a source of genomic instability, leading to DNA breaks, rearrangements and recombination [169], [170], [171] [R-loops reviewed in [172], [173], [174]].

The formation of R-loops and G4s is correlated, where one structure may facilitate the formation of the other. If the displaced ssDNA loop within an R-loop is G-rich, it can fold into a G4; the resultant structure is called a G-loop [118], [175]. Chemical stabilisation of G4s has also been shown to enhance R-loop formation [176], [177]. Furthermore, genome-wide profiling and mapping approaches in living cells have confirmed their co-localisation [176], [178]. G-loops may play a physiological role in gene expression and genome organization via CTCF (Transcriptional repressor CTCF) [173], [179]. Importantly, since G-loops contain secondary structures on both DNA strands, they are most likely potent barriers to replication fork progression and have been shown to contribute to genomic instability in cancer [118].

#### 1.2.6 Z-DNA

Z-DNA is a left-handed form with a characteristic zig-zag-shaped backbone, arising in sequences of alternating purines and pyrimidines [180]. Z-DNA's cellular roles include gene expression regulation, and inflammatory cell death and type I interferon production during viral infection [181], [182]. Z-DNA can induce torsional stress and cause DSBs, leading to large-scale deletions [183], [184]. However, while replication may stabilise Z-DNA by supplying torsional stress [185], there is limited direct evidence for Z-DNA as a replication-fork barrier, therefore we do not discuss Z-DNA further [reviewed in [181], [186], [187]].

### 1.3 Resolution of DNA secondary structures

Given that DNA secondary structures can impede fork progression, many cellular mechanisms have evolved to help replicate these loci and thus safeguard genome stability. If a structure is weak or transient, replisome-intrinsic activities can rescue the stall. For example, Pol δ can use its strand displacement activity to resolve hairpins in vitro [223]. If a structure is stable, forks rely on extrinsic factors to rescue the stall. We propose that this occurs as a tiered response, based on when and where the structure appears relative to CMG. RPA is the first responder, which can prevent folding, recruit rescue proteins and instigate checkpoint responses. This is followed by structure-selective accessory helicases that can unfold persistent structures. Finally, tolerance pathways, such as fork reversal, fork protection, repriming and TLS, can protect the fork while allowing fill-in and/or bypass of any nicks/gaps. Comprehensive mechanisms of resolution are available in recent reviews [224], [225], [226].

#### 1.3.1 RPA and accessory helicases

RPA, comprised of RPA70, RPA32 and RPA14 subunits [227], responds to a stall by coating newly exposed ssDNA, which both seeds ATR signalling and creates a tolerance window before collapse [228], [229]. RPA can also aid in structure resolution. Single-molecule and biochemical work shows that RPA can both prevent G4 formation and unfold a subset of G4s, with efficiency shaped by G4 topology [230], [231], [232], [233], [234]. RPA can transiently melt or suppress hairpins by diffusing in from adjacent ssDNA [235]. Single-molecule FRET demonstrates RPA binding and remodelling of iMotifs in vitro [236]. Despite this evidence, high concentrations of RPA were unable to allow reconstituted budding yeast replisomes to replicate past stable CG-rich hairpins, G4s and iMotifs [223]. This suggests that RPA may only facilitate replication of relatively unstable/weak structures. Furthermore, direct locus-specific in vivo evidence for RPA’s role at structure-stalled replication forks remains limited [reviewed in [237], [238]].

In response to replication stress, ATR rapidly phosphorylates RPA32 at Serine 33 on RPA-ssDNA, initiating checkpoint signalling and stabilising the fork [239], [240]. Under more extensive or DSB-like replication stress, DNA-PK (DNA-dependent protein kinase)-dependent Serine 4/8 hyperphosphorylation engages and modulates checkpoint intensity and fork restart pathway choice [241]. In G2, Ser4/Ser8 helps sustain ATR-CHK1 signalling by promoting Rad9/TOPBP1 (Cell cycle checkpoint control protein RAD9A/DNA topoisomerase II binding protein 1) assembly [242]. At secondary-structure loci, the G4-stabilising ligand telomestatin elevates RPA32-Ser33 phosphorylation in human glioma stem cells, accompanied by TRF2 (Telomeric repeat-binding factor 2) loss from telomeres and CHK1 activation; pSer33 foci form in a replication/transcription-dependent manner, while matched non-stem glioma cells show pS33 without robust checkpoint engagement [243].

Eukaryotes encode dozens of DNA helicases (SF1-SF6) [244], [245], [246]. The replicative helicase CMG (MCM2–7) belongs to the SF6 superfamily, whereas most structure-directed accessory helicases belong to the SF1/SF2 superfamilies, including PIF1 [247] and DNA2 (DNA replication helicase/nuclease 2) [248]; DEAH-box family helicase DHX36 (DEAH-box helicase 36)/RHAU [249]; iron-sulfur cluster (Fe-S) family helicases FANCJ (Fanconi anemia complementation group J)/BRIP1, RTEL1 (Regulator of telomere elongation helicase 1), and DDX11 (DEAD/H-box helicase 11)/ChlR1 [250], [251]; and RecQ family helicases BLM (Bloom Syndrome protein), WRN (Werner Syndrome protein), RECQL1 (RecQ protein-like 1), RECQL4 (RecQ protein-like 4), and RECQL5 (RecQ protein-like 5) [250], [252], [253] (Table 1). Most of the mechanistic details of these helicases to date is derived from G4 studies [reviewed in [224], [254], [255]].

**Table 1:** Resolution of DNA secondary structures by accessory helicases.

**Helicase** **Directionality**	**DNA secondary structure resolution**	**Replisome-specific** **interactome**	**Relationship with other helicases**	**Clinical Relevance**
**PIF1** [scPif1/spPfh1] **5’-3’**	**Hairpin** In vitro [223], [282], [283] **G4** In vivo [128], [129], [264], [284], [285], [286], [287], [288], [289], [290], [291] In vitro [247], [282], [283], [292], [293], [294], [295], [296], [297] **iMotif** In vitro [223], [295] **R-loop** In vivo [298] In vitro [296]	RPA [265] PCNA [129] CMG [299] Replicative polymerases [299]	DNA2 [300]	Breast/kidney cancer risk/prognosis [301], [302] BRCA2 null cancers [141]

**DNA2** [scDna2] *Helicase and nuclease activity **5’-3’**	**G4** In vitro [266], [303]	RPA [266]	PIF1 [300] BLM [271], [304], [305], [306], [307] WRN [304], [306], [308], [309]	Seckel syndrome and microcephalic primordial dwarfism [310], [311]

**DHX36** (G4R1/RHAU) **3’-5’**	**G4** In vivo [256], [312], [313], [314] In vitro [315], [316], [317], [318], [319]	RPA [312]	FANCJ [256]	Neurodegeneration [249] Prognostic marker in lung cancer [320] Cardiomyopathy in mice [313]

**FANCJ** (BRIP1/BACH1) [*C.elegans dog-1*] **5’-3’**	**Hairpin** In vivo [321] In vitro [322], [323] **Triplex** In vivo [321] In vitro [324] **G4** In vivo [111], [256], [257], [261], [325], [326], [327], [328] In vitro [322], [323], [326], [329], [330], [331], [332], [333] **5’-flap DNA (Okazaki intermediate)** In vitro [334]	RPA [335] PCNA [329], [330] AND-1 [336]	BLM/WRN [261], [329], [337], [338] DHX36 [256] RTEL1 [339] DDX11 [257], [258]	Fanconi Anaemia [321], [330] Breast/ovarian cancer predisposition [340]

**RTEL1** **5’-3’**	**G4** In vivo [262], [263], [275], [277] In vitro [276], [341], [342] **Overlapping G4/R-loop**[275], [277]	RPA [276] PCNA [343] CMG and Pol ε [344] MCM10 [341]	BLM [262], [263]	Hoyeraal-Hreidarsson Syndrome [345] Dyskeratosis Congenita [346]

**DDX11** (Chlr1) [scChl1] **5’-3’**	**Triplex** In vitro/In vivo [188] **G4** In vivo [257], [258] In vitro [347] **5’-flap DNA (Okazaki intermediate)** In vitro [348]	PCNA [349] Pol δ [350] TIMELESS [347], [351] FEN1 [188], [349], [351] AND-1/Ctf4 [352]	FANCJ [257], [258]	Warsaw Breakage Syndrome [347], [353]

**BLM** (RECQ2/RECQL3) [scSgs1] **3’-5’**	**Hairpin** In vitro [354] **Triplex** In vitro [355] **G4** In vivo [259], [260], [280], [356], [357], [358], [359] In vitro [360], [361], [362], [363], [364], [365], [366], [367], [368] **5’-flap DNA (Okazaki intermediate)** In vitro [369], [370] **Overlapping G4/R-loop** In vivo [178]	RPA [227], [267], [272], [273] Pol δ [371] MCM6 [372] FEN1 [369]	DNA2 [271], [304], [305], [307] WRN [260], [305], [306], [373], [374], [375] FANCJ [261], [337], [338] RTEL1 [262], [263] RECQ4 [376]	Bloom’s Syndrome [377], [378]

**WRN** (RECQ3/RECQL2) *Helicase and exonuclease activity **3’-5’**	**Hairpin/Cruciform** In vivo [65] In vitro [308], [379] **Triplex** In vitro [355] **G4** In vivo [259], [260], [357], [380], [381] In vitro [361], [363], [368], [382], [383], [384] **5’-flap DNA (Okazaki intermediate)** In vitro [369], [385] **Overlapping G4/R-loop** In vivo [178]	RPA [270], [272], [386] PCNA [309], [387] Pol δ [308], [388], [389], [390] FEN1 [267], [369]	DNA2 [304], [308], [309] FANCJ [261] BLM [260], [305], [306], [373], [374], [375] RECQ1 [391] RECQ5 [392]	Werner’s Syndrome [393], [394]

**RECQ1** (RECQL1) **3’-5’**	**G4** In vitro [395], [396]	RPA [397] PCNA [398] FEN1 [399]	WRN [391]	RECON Syndrome [400], [401]

**RECQ4** (RECQL4) **3’-5’**	**G4** In vivo [402] In vitro [403], [404]	RPA [404] MCM2–7 [405] AND-1/Ctf4 [406] MCM10 [406] Pol α [407] MCM10 [405], [408]	BLM [376]	Rothmund-Thomson, RAPADILINO and Baller-Gerold Syndromes [409]

**RECQ5** (RECQL5) **3’-5’**	**G4** In vitro [410]	PCNA [411], [412]	WRN [392]	

Summary of evidence of accessory helicase implicated in the resolution of DNA secondary structures, Superfamily 1 (PIF1, DNA2). Superfamily 2: DEAH-BOX (DHX36); SF2 Iron-Sulfur Cluster (FANCJ, RTEL1, DDX11); SF2 RECQ (BLM, WRN, RECQ1, RECQ4, RECQ5). Names include common aliases (in parentheses) and, where relevant, orthologues from other organisms (in brackets), as the cited studies span multiple species. Columns indicate polarity, substrates, interactions with replisome components, relationships with other helicases, and human syndromes or tumour dependencies linked to loss of function.

Abbreviations: PIF1 (ATP-dependent DNA helicase PIF1/Petite Integration Factor 1); DNA2 (DNA replication helicase/nuclease 2); DHX36 (DEAH-box helicase 36); FANCJ (Fanconi anemia complementation group J); RTEL1 (Regulator of telomere elongation helicase 1); DDX11 (DEAD/H-box helicase 11); BLM (Bloom Syndrome protein); WRN (Werner Syndrome protein); RECQL1 (RecQ protein-like 1); RECQL4 (RecQ protein-like 4); RECQL5 (RecQ protein-like 5); sc (*Saccharomyces cerevisiae); sp (Schizosaccharomyces pombe); C. elegans (Caenorhabditis elegans).*

Interestingly, these helicases have been shown to act in overlapping and partially redundant pathways to resolve G4 structures in vivo. DHX36 and FANCJ cooperate for the unwinding of leading strand G4s and also for G-loop disassembly at transcribed loci [175], [256]. FANCJ also contributes alongside RTEL1 to limit G4 accumulation in cells [201]. Moreover, FANCJ and DDX11 act in genetically separable, partially redundant, G4-resolving pathways, with DDX11 dominating the response to certain G4 ligands and FANCJ acting on a distinct subset of G4s [257], [258]. BLM and WRN provide parallel RecQ helicase activities at G4 motifs [259], [260] and cooperate with FANCJ to maintain epigenetic stability [261]. At telomeres, RTEL1 and BLM act in partly separate pathways to promote replication through telomeric G4 DNA and suppress fragile telomeres [262], [263]. Overall, most evidence comes from G4 DNA, but it remains unclear how strand orientation, structure type and topology, chromatin context and replication timing determine when particular helicases are specifically required versus redundant during replication, or whether similar principles apply to other DNA structures. Moreover, beyond these canonical helicases, a recent study identified the DEAD-box RNA helicase DDX3X (ATP-dependent RNA helicase DDX3X) as a triplex-binding factor with ATP-independent triplex-unwinding activity [189], suggesting that additional factors may also contribute to resolving other secondary structures. For a summary of the relationship between helicases in other cellular pathways see Table 1.

RPA-ssDNA regulates various accessory helicases at stalled forks. This hierarchy is exemplified at telomeres: RPA acts as a first-line ssDNA chaperone to prevent G4 formation on lagging strand telomeres, with scPif1/spPfh1 providing G4-unwinding back-up [264], [265]. RPA and DNA2 cooperate to process G4s [266] and can resolve long/structured flaps in OFM in vitro [248]. Moreover, RPA binds and stimulates both BLM and WRN helicase activity [267], [268], [269], [270], [271] with mapped RPA-binding domains in BLM/WRN [227], [272], [273]. In cells, the RPA-BLM interaction is required for fork restart [273] and the RPA-WRN interaction promotes fork recovery after replication stress and limits G4 persistence [274]. Interestingly, high RPA loading drives WRN into a markedly hyper-processive state [270]. An additional RPA-centred layer is provided by HERC2 (HECT and RLD domain containing E3 ubiquitin protein ligase 2), which bridges BLM and WRN with RPA to suppress G4s in cells [260]. An RTEL1-RPA interaction operates at G-loop loci, consistent with roles at telomeres and TRCs [275], [276], [277]. Thus, RPA is indispensable for the action of some accessory helicases.

Mutations in many accessory helicases cause human disease and sensitise genomes to structure-derived instability (Table 1): *dog-1*/FANCJ deficiency causes G-tract deletions in *Caenorhabditis elegans* [278]; RTEL1 suppresses trinucleotide-repeat expansion and associated fragility [279]; BLM loss elevates recombination at G4 and fragile regions [280], [281]; and WRN is essential in tumours burdened with microsatellite instability (MSI) due to expanded cruciform-forming (AT)_n_ repeat tracts [65]. Together, these data highlight the important roles that accessory helicases play in normal human physiology, and support a model in which helicase specificity, ordered cooperation, and fork-proximal recruitment are central to preventing secondary structure-induced replication failure.

#### 1.3.2 Polymerases

While helicases can unfold secondary structures, polymerases are essential to resume synthesis. Pol δ can utilise its strand displacement activity for structure resolution of hairpins [223], [413] [reviewed in [414]] (Table 2). Genetic evidence in fission yeast indicates that Pol δ proofreading activity helps reinitiate synthesis after fork stalling [415] and its disease-linked mutations underscore its relevance [414]. TLS polymerases, including REV1 (DNA repair protein REV1), Pol η (DNA polymerase eta), and Pol κ (DNA polymerase kappa) and Pol ζ (DNA polymerase zeta catalytic subunit), are primarily implicated in the tolerance of damaged templates. However, there is evidence that they can also resolve short-lived structure-induced stalls. REV1 can destabilise/unfold G4s and enable limited bypass, restraining persistent obstruction and breakage [416], [417] (Table 2), and may also target triplexes [418], [419]. TLS polymerases can also extend across various structures: Pol η extends across parallel G4s and triplexes [420], [421]; Pol κ extends across G4s, triplex-forming repeats [420], [421]; and Pol ζ extends across hairpins [422] [reviewed in [423], [424], [425], [426]]. Interestingly, it has been shown in vitro that G4 sequence and topology modulates the fidelity of human Pol δ, κ and η, with parallel G4 motifs causing more local deletions, insertions and frameshifts than antiparallel or hybrid G4s [427]. If stable structures persist, PrimPol (DNA-directed primase/polymerase protein) can reinitiate synthesis on exposed ssDNA downstream of the block [428], [429]. PrimPol-mediated repriming is a major route for bypass of leading and lagging strand G4 arrays in vertebrate cells [430], [431], [432] [reviewed in [224], [423], [425]].

**Table 2:** Resolution of DNA secondary structures by polymerases.

**Type**	**Polymerases**	**Structure targeted**	**Mechanism**	**Interactions with replisome/helicases**	**In vivo significance** **(structure-specific)**
**Replicative polymerase**	**Pol δ**	**Hairpin** [223]	3’ to 5’ exonuclease activity in overcoming replication obstacles [414] Strand displacement activity enables structure unwinding [413] Topology-dependent error signatures during G4 synthesis in vitro [427]	PCNA [433], [434] FEN1 [435], [436] Pol alpha [437], [438] RPA [439] BLM [371] WRN [308], [388], [389], [390] DDX11 [350]	

**TLS polymerase**	**REV1**	**G4** [416] **Triplex** [418], [419]	Physically disrupts G4; inserts dCMP; hands to Pol ζ [416], [417]	PCNA [440], [441] FANCJ [330]	Required at G4 loci for fork progression/chromatin maintenance [123], [261], [442]
	**Pol η**	**G4** [420], [421], [443] **Triplex** [420], [421]	High-fidelity extension across parallel G4/triplex [420], [421], [424] Topology-dependent mutational signatures during G4 synthesis [427]	Genetic link with PIF1 and FANCJ [261], [284], [442] PCNA [444], [445] WRN [446]	Increased sensitivity to G4-stabiliser when depleted [443]
	**Pol κ**	**G4** [420], [421] **Triplex** [443]	Rapid, error-prone synthesis 2 nt before the first G4 tetrad [421] Induces frameshift errors within G-tracts of parallel G4 motifs in vitro [427]	REV1 [445], [447], [448] PCNA [444], [449] WRN [446], [450]	
	**Pol ζ**	**Hairpin and cruciform** [422]	Extension after REV1/η/κ insertion [416], [417], [421], [451]	PCNA [440]	Suppresses deletions at hairpin-rich loci [422], [442]

Replicative and tolerance polymerases that synthesise through or around DNA secondary structures. Columns indicate polymerase class, structure targeted, dominant mode of action, interactions with fork factors and in-cell significance, which highlights cellular phenotypes/genetic dependencies.

In summary, current data supports a layered response in which RPA coats ssDNA to suppress refolding and organises hand-off to accessory helicases and tolerance pathways. Helicases promote CMG bypass and/or directly unwind structures, while Pol δ uses strand displacement to remodel hairpins/flaps and re-establish synthesis. If synthesis is still delayed, REV1 can initiate TLS or destabilise compact G4s while PrimPol provides resumption of synthesis at the cost of leaving a gap. Altogether, these pathways support fork progression through DNA secondary structures.

## 2 PART 2: structure-induced replication fork instability

### 2.1 Secondary structures as fork barriers

**Fig. 3:**
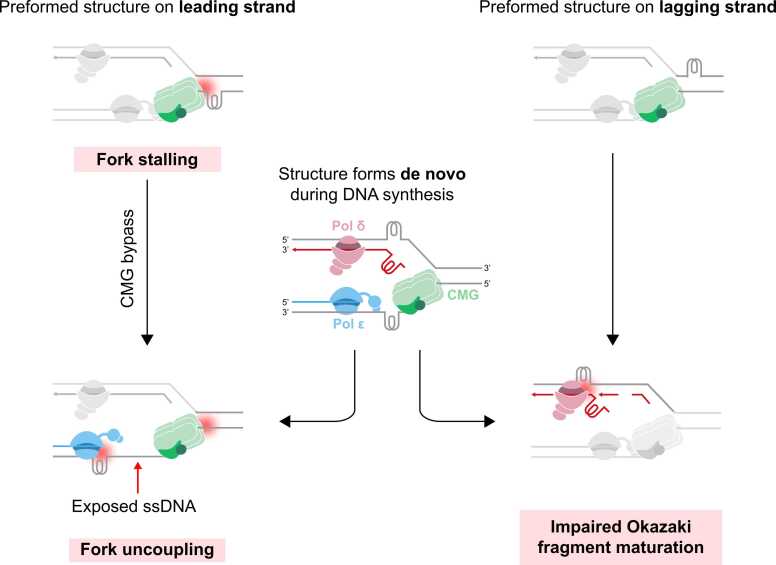
DNA secondary structures as barriers to fork progression. Outcomes depend on where the structure lies (leading vs lagging template) and when it forms (pre-formed vs de novo). Left: A pre-formed structure on the leading strand template is encountered by CMG first, causing fork stalling. If CMG bypasses, the structure will be encountered by Pol ε, leading to fork uncoupling and exposure of ssDNA. During fork uncoupling, CMG is also impacted as its unwinding speed is reduced. Centre: Structures can form de novo during replication behind the CMG on either leading or lagging strands. Such de novo structures will be encountered by the leading and lagging strand synthesis machinery, leading to either fork uncoupling or impaired Okazaki fragment maturation respectively. Right: A pre-formed structure on the lagging strand template leaves CMG largely unaffected but impairs Okazaki fragment maturation either on the template or the nascent lagging strand. These immediate fork states, uncoupling on the leading template and maturation defects on the lagging template, are early intermediates associated with genome instability if left unresolved. Abbreviations: CMG, CDC45-MCM-GINS helicase; ssDNA, single-stranded DNA; Pol ε, DNA polymerase epsilon; Pol δ, DNA polymerase delta.

To date, primer extension assays and biochemical studies using templates with structure-forming sequences have established that hairpins, triplexes, G4s and iMotifs can stall synthesis by prokaryotic and eukaryotic DNA polymerases [223], [422], [452], [453], [454], [455], [456], [457], [458], [459], [460], [461], [462]. Importantly, it has become evident that the severity of polymerase stalling is influenced by thermal stability and topology. For instance, G4s with higher thermostabilities correlate with stronger inhibition of synthesis [459], [463], [464]. More recent single-molecule and structural studies support these findings [256], [295], [465], [466]. Hence, a variety of data suggests that DNA secondary structures act as barriers to fork progression [reviewed in [138]]. How a secondary structure perturbs replication depends on when it forms (pre-formed vs de novo during replication) and where it appears (leading vs lagging strand template). These parameters define the first point of contact (CMG or replicative polymerases) and biases the fork towards certain outcomes such as fork stalling, fork uncoupling, or OFM impairment (Fig. 3).

#### 2.1.1 CMG helicase progression

CMG is the first point of contact and will only encounter DNA secondary structures if they are pre-formed i.e., present before replication fork arrival. Pre-formed structures can be produced in a variety of ways (e.g. supercoiling and ssDNA exposure during transcription or DNA repair) and can also cause other issues outside of replication (e.g. transcriptional impairment) [467], [468], [469]. Furthermore, only leading strand pre-formed structures would act as CMG barriers since the lagging strand is excluded from CMG (Fig. 3, left). Such structures have been studied with the use of engineered inserts or topologically favoured extrusions; all are shown to stall CMG progression [256], [281]. For instance, in *Xenopus laevis* egg extracts, a pre-formed leading strand G4 stalled CMG progression, whereas a lagging strand G4 did not [256]. Using a reconstituted budding yeast replisome, it was also shown that a pre-existing leading strand G4, generated by an R-loop, could block CMG progression even if the R-loop was first resolved [465]. Cryo-electron microscopy (cryo-EM) reconstitution of a G4 and CMG provided atomic-level evidence that DNA shape alone can stall the replisome [466]. In this structure, a pre-formed leading strand G4 is internalised by CMG and sits at the N-/C-tier interface, being too bulky to pass through the central channel [466]. The G4 blocks the PS1 and H2I hairpins within the AAA+ ATPase C-tier motor of CMG. Interestingly, the overall architecture of the G4-arrested fork, including the lagging strand template path and overall DNA-protein contacts, are essentially identical to an unperturbed fork. This suggests that a G4-arrested CMG would not be detected as an aberrant intermediate while sequestering the G4 away from G4-unwinders [466].

Some studies have also shed light on how CMG can bypass obstacles. Work from the Knipscheer group showed that DHX36 creates a short run of leading strand ssDNA ahead of CMG, which allows CMG to slide past intact G4s [256]. This mechanism is reminiscent of the ability of RTEL1 to facilitate CMG bypass of DPCs by generating downstream ssDNA [470]. The idea that CMG might “skip” over obstacles is supported by single-molecule imaging and biochemical work, showing an MCM10-dependent ring opening mechanism that enables CMG to transition between ssDNA and dsDNA binding modes [471]. Additionally, in reconstituted CMG unwinding assays, placing a G4 on the leading strand enforces CMG pausing with eventual slow bypass [295], [465]. Therefore, DNA secondary structures may only stall CMG temporarily, with bypass enabled either by accessory helicases, ring opening, or both.

The molecular features of secondary structures enable us to make informed predictions about their effect on CMG progression. For instance, one can envision that structures formed via canonical Watson-Crick base pairing, such as hairpins, could directly be unwound by CMG, thereby having no effect on CMG progression. However, there is no evidence for this yet. Additionally, it can be mechanistically useful to benchmark secondary structures against other types of obstacles, such as interstrand crosslinks (ICLs), DNA-protein crosslinks (DPCs), and UV photoproducts (e.g., cyclobutane pyrimidine dimers (CPDs)). For instance, leading strand DPCs strongly stall CMG [472], [473], [474] allowing us to speculate about the impact of secondary structures on CMG progression, albeit with caution given the dynamic nature of these structures. The size and structure of a protein within a DPC dictates the efficiency of CMG bypass [475]. This is because the CMG helicase ring is proposed to open to bypass the DPC, with larger DPCs being naturally harder to traverse [475].

By analogy, those structures that cannot be unwound would eventually be bypassed by CMG, with the size, shape and stability of the structure likely dictating bypass efficiency. It is conceivable that more stable structures such as G4s with short loops [93] or tightly stacked triplexes, are more likely to require active mechanisms such as generating ssDNA downstream and/or MCM ring opening for CMG bypass. Alternatively, it is also possible that CMG can unwind structures formed via Hoogsteen base pairing, such as G4s and iMotifs, however with lower efficiency.

Certain structures might block CMG more severely, leading to complete fork arrest. This would be analogous to ICLs, which prevent strand separation and arrest CMG on first encounter even in wild-type cells [5], [476]. In contrast, CPDs and other small leading strand obstacles do not affect CMG progression, and rather prevent pol ε synthesis [473], [474], [477]; therefore, structure size may be an important determinant in CMG stalling. The strand orientation is also important: lagging strand DPCs typically do not block CMG. Therefore, lagging strand structures are also unlikely to obstruct CMG [5], [472], [474], [478].

Non-covalently bound proteins also act as weaker obstacles to fork progression. Artificial examples include operator-repressor arrays such as LacO/LacI and TetO/TetR, which slow forks without definitive CMG arrest, whereas the prokaryotic Tus-Ter barrier imposes stronger fork stalling and can provoke breakage when present in arrays [479], [480], [481], [482], [483]. Endogenous DNA-binding proteins, such as telomeric proteins, can also convert a permissive sequence into a helicase and polymerase block [484]. Thus, although the dynamics and downstream responses differ, we can draw parallels in the early events in CMG encounter (either stalling or eventual bypass) between different replication obstacles.

#### 2.1.2 Leading strand synthesis: Pol ε

The leading strand polymerase, Pol ε, can encounter DNA secondary structures in two scenarios. First, when the CMG helicase bypasses a pre-formed leading strand structure (Fig. 3, left), and second, when a de novo structure arises during replication within exposed ssDNA between CMG and Pol ε (Fig. 3, centre). However, it is unclear whether there would be sufficient ssDNA between CMG and Pol ε for secondary structure formation. During normal fork progression, the CMG leading strand exit channel delivers the template leading strand directly to Pol ε [5], [13], [485], [486]. Such proximity would leave little accessible ssDNA, rendering de novo folding unlikely. Alternatively, Pol ε may undergo a dynamic conformational switch which could alter the ssDNA length at this interface. Such movement is explained by the structure of Pol ε: it is bound to CMG via its non-catalytic domain, and its catalytic domain is flexibly tethered [13], [487]. Given this flexibility, current structural studies cannot resolve the ssDNA in the gap between CMG and Pol ε, so its precise length goes unreported. Single-molecule studies suggest that CMG-bound Pol ε dynamically exchanges with soluble Pol ε, which provides another route for ssDNA exposure [488]; whether or how frequently this occurs in vivo is uncertain. Since coupling leading strand synthesis to unwinding helps prevent structure formation, scenarios that induce uncoupling increase the probability of structure formation. These include general replication stress (e.g. nucleotide depletion), canonical DNA obstacles (e.g., CPDs) or DNA secondary structures themselves [295], [473], [474], [489].

Evidence that secondary structures can form de novo during replication comes from studies of a fully reconstituted budding yeast replisome, where a poly(dG)_16_ or poly(dC)_40_ tract on the leading template can fold de novo into G4s or iMotifs, respectively, and (CG)_n_ and (CGG)_n_ repeats form replisome-stalling hairpins [223], [295]. Such structures on the leading strand trigger fork uncoupling, whereas the same tracts on the lagging strand do not [223], [295]. Importantly, Williams et al. used solid-state nanopores to demonstrate that neither the poly(dG)_16_ nor the poly(dC)_40_ substrate contained pre-folded structures prior to replication [295]. These results are consistent with super-resolution imaging of G4s and replisomes in cells, demonstrating that G4s are primarily located behind CMG, although in this approach one cannot distinguish between de novo structures and bypassed ones [111].

Regardless of whether they form de novo or are bypassed by CMG, DNA secondary structures can block synthesis by Pol ε, leading to fork uncoupling. CMG continues unwinding at ∼15–30 % of its normal speed, leading to exposure of ssDNA (Fig. 3, bottom left). In fission yeast, G4 stabilisation elevates RPA-ssDNA and impedes replication [490]. Super-resolution imaging in human cells detects an increase of G4s at active forks under mild polymerase inhibition (aphidicolin (APH)) and with G4 stabilisation RPA rises globally yet remains locally constrained at G4-replisomes unless FANCJ is present [111].

#### 2.1.3 Okazaki fragment synthesis and maturation

The natural asymmetry of the replication fork means that there are inherent differences between the leading and lagging strand. Lagging strand synthesis is discontinuous and therefore inherently more tolerant to strand impediments, as priming downstream by Pol α occurs efficiently. Furthermore, obstacles on the lagging strand do not affect CMG or Pol ε activity and vice versa; lagging strand synthesis continues unaffected when synthesis is inhibited on the leading strand [473], [474]. However, CMG has been shown to temporarily stall when it encounters lagging strand DPCs that stabilise duplex DNA [472], [478]. In contrast, it is unlikely that DNA secondary structures on the lagging strand will stabilise duplex DNA and therefore may not affect CMG progression. The lagging strand machinery can encounter pre-formed or de novo secondary structures (Fig. 3, centre and right). De novo structures can either form on the template strand itself, or on 5’ ssDNA flaps generated during strand displacement; structure formation is more likely on longer flaps. In line with this, in vitro and cellular studies have shown that G4- or triplex-forming motifs in the lagging strand template can stall fork progression by impacting lagging strand synthesis, and not CMG progression per se [287], [465], [466]. In addition, hairpins and triplex-forming repeats on model 5’ flap substrates are poor substrates for FEN1 cleavage [491], [492], [493]. At inverted repeats, replication-dependent lagging strand ssDNA hairpins are proposed to predominantly mediate fork stalling in vivo in budding yeast [52], and in *E. coli* interrupted palindromes generate lagging strand hairpins each replication cycle [51], [53].

In summary, strand context determines both folding propensity and outcome. On the lagging strand, transient ssDNA or 5′ flaps favour de novo folding and affect OFM [129], whereas leading strand folds confront CMG and/or Pol ε [123], [128], [223], [295]. Asymmetry is also enzymatic - recent primer extension assays revealed that human Pol δ and Pol ε exhibit different levels of inhibition by a range of G4 structures [464]. In addition, Pol δ can rescue leading strand stalls induced by hairpin-forming repeats, most likely via its strong strand displacement activity relative to Pol ε [223]. Overall, leading strand structures may occur less frequently due to helicase-polymerase coupling but will be more intrinsically toxic (Fig. 3), whereas lagging strand structures may occur more frequently but are more easily bypassed, typically manifesting as gaps or OFM defects (Fig. 3).

In the next sections, we will outline how structure-induced fork stalling and uncoupling can cause adverse consequences, primarily the introduction of ssDNA gaps and DNA breakage. We will frame such models by explaining the factors that affect the pathway taken to result in such damage, beginning with the roles of RPA and the S phase checkpoint.

### 2.2 RPA and the S phase checkpoint

Stable secondary structures encountered on the leading strand template can drive fork uncoupling, exposing ssDNA that is rapidly coated by RPA, which prevents structure refolding and scaffolds helicases and tolerance factors (Section 1.3). Moreover, RPA-ssDNA engages ATR-ATRIP (ATR-interacting protein), thereby acting as the local entry point for checkpoint control [reviewed in [21], [25], [228]]. The RPA-ssDNA platform also recruits fork response factors that promote either fork reversal or repriming (see Section 2.3)*.* Phosphorylation of RPA further tunes this hub, modulating PALB2/BRCA2 (Partner and localiser of BRCA2/Breast cancer type 2 susceptibility protein) engagement and RAD51 (DNA repair protein RAD51 homolog 1) loading to protect or restart stalled forks [228], [494]. Thus, RPA and the S phase checkpoint are intrinsically intertwined. The ATR-CHK1 S phase checkpoint (and its functional equivalents, Mec1-Rad53 and Rad3-Cds1 in budding and fission yeast, respectively) creates a window in which stalled or uncoupled forks can be remodelled or bypassed by restraining origin firing to prevent RPA exhaustion and by directly limiting fork uncoupling via checkpoint control of CMG [242], [495], [496], [497]. Consistent with this, human cells can tolerate substantial ssDNA provided RPA is sufficient [229] and ATR inhibition induces fragility at structure-forming repeats and long poly(dA:dT) tracts [495], [498], [499].

Reconstitution work clarifies how checkpoint kinases moderate CMG unwinding and stabilise the replisome. In budding yeast replication systems, Rad53 (CHK2) limits CMG unwinding and uncoupling under stress [496] and phosphorylates Mrc1 (CLASPIN) and Mcm10 to reduce fork uncoupling [497]. Under dNTP depletion in *Saccharomyces cerevisiae*, Rad53 activity limits fork uncoupling and re-couples leading and lagging strand synthesis, thereby reducing ssDNA [496], [497], [500]. In *Schizosaccharomyces pombe*, the Rad53 homolog Cds1 (DNA replication checkpoint kinase Cds1) phosphorylates CDC45 to slow CMG under hydroxyurea (HU), preventing fork uncoupling [501]. Additional *Saccharomyces cerevisiae* work details how Rad53 maintains replisome integrity under replication stress [502].

A key operational detail for tolerance of replication stress is PCNA cycling, which is coupled with OFM. PCNA is usually unloaded after an Okazaki fragment is fully processed and ligated [503], [504], [505], [506]. If a nick or gap remains, PCNA remains bound to support further processing. During uncoupling, lagging strand synthesis continues, and incomplete Okazaki fragments sequester PCNA and RFC; checkpoint-mediated restraint of CMG progression minimises further uncoupling on the leading strand while limiting lagging strand fragment accumulation. This preserves PCNA/RFC pools and stabilises forks until the obstacle is tolerated or removed [507], [508]. In human cells, checkpoint signalling limits excessive Okazaki fragment accumulation and prevents depletion of PCNA, thereby protecting forks from collapse and preserving tolerance or restart capacity [507], [508].

### 2.3 How secondary structures destabilise the fork: proposed models

In this section, we focus on fork-borne intermediates generated by secondary structures, how these are processed into ssDNA gaps and how they might yield DSBs within the same S phase. We distinguish these from the downstream genetic consequences of such damage that record how those intermediates were eventually resolved.

DNA secondary structures are handled by the same core pathways as other obstacles [48], [509]. Because mechanisms linking replication of secondary structures to resultant gaps and breaks remain poorly defined, we ground the discussion in established replication stress principles. However, the dynamics and context of structures distinguish them from other challenges, such as damaged DNA or DPCs, which demand chemical removal or proteolysis before synthesis can continue.

#### 2.3.1 Model 1: fork arrest

A stable secondary structure on the leading strand would be encountered first by CMG (Fig. 3). Such stable structures could arrest CMG severely, potentially shielding the structure from accessory helicases if lodged within the central channel, as observed for G4s [466]. Although the outcome of an arrested CMG is not clear, we can make informed assumptions, as outlined below.

**Fig. 4:**
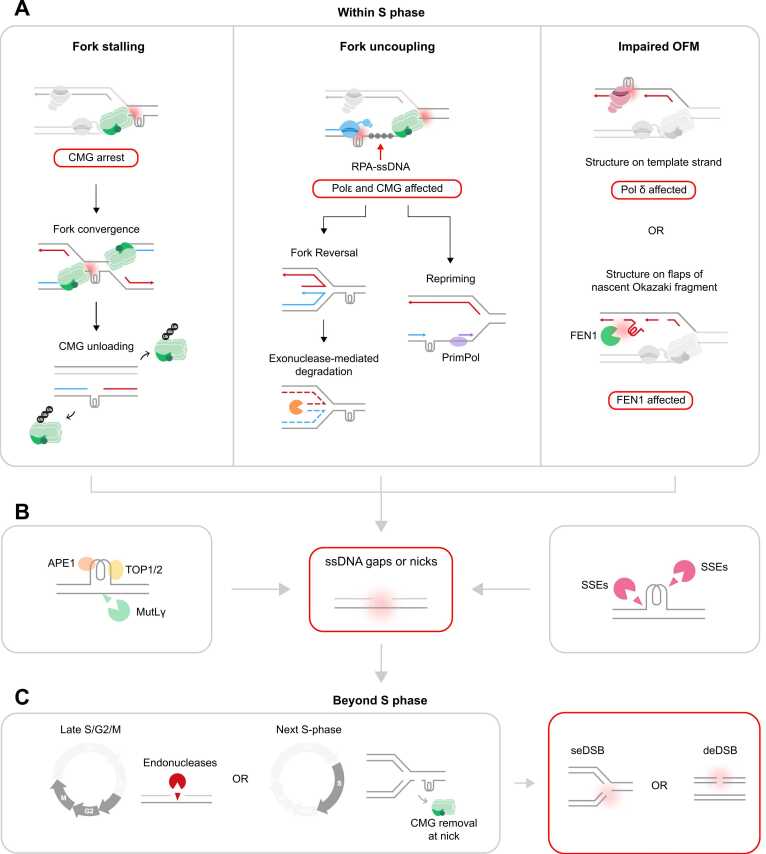
Proposed models linking DNA secondary structures to gaps and breaks. (A) Immediate consequences: We propose three major immediate outcomes when a fork encounters DNA secondary structures. Left: Model 1: Stable secondary structures on the leading strand cause CMG arrest, leading to fork stalling. Assuming a long-lived arrest, the most likely outcome is fork convergence and CMG unloading, generating ssDNA gaps on the leading strand. Centre: Model 2: Secondary structures on the leading strand can also lead to fork uncoupling, whereby Pol ε and CMG will be impacted, leading to formation of RPA-coated ssDNA stretches. This can either trigger fork reversal (Model 2 A) or PrimPol-mediated repriming (Model 2B). Mis-regulation of exonucleases at reversed forks can lead to uncontrolled nascent strand degradation, thereby generating ssDNA gaps. PrimPol-mediated repriming without gap filling can also lead to formation of ssDNA gaps. Right: Model 3: De novo secondary structures on the lagging strand template impact DNA synthesis by Pol δ (Model 3 A), whereas nascent lagging strand flap structures impact OFM factors such as FEN1 (Model 3B). Both scenarios lead to lagging strand gaps or nicks. (B) Other routes to gaps or nicks: Gaps or nicks can also arise from cleavage and processing by various components, such as APE1, which might also be influenced by APOBEC3 deamination (not shown), TOP1/2, MutLγ, and other structure-specific endonucleases (SSEs). (C) Beyond S phase: Gaps or replication intermediates that escape repair in late S/G2 phase can break in G2 or mitosis due to SSEs or other mitotic endonucleases*.* Gaps or nicks that persist until the next S phase will lead to fork collapse. Both scenarios can yield either a single-ended DSB (seDSB) or a double-ended DSB (deDSB) (see Box 2 for details). Abbreviations: CMG, CDC45-MCM-GINS helicase; OFM, Okazaki fragment maturation; FEN1, flap endonuclease-1; PrimPol, DNA primase-polymerase; ssDNA, single-stranded DNA; TOP1/2, topoisomerases I/II; MutLγ, MLH1-MLH3 mismatch-repair endonuclease; SSE(s), structure-selective endonucleases (e.g., MUS81-EME1, SLX1-SLX4, XPF-ERCC1); seDSB, single-ended double-strand break; deDSB, double-ended double-strand break.

If CMG arrest persists for a long period, a converging fork will eventually arrive and trigger CMG removal. At canonical termination, CMG unloads when it no longer engages the lagging strand template, thereby exposing MCM7 for ubiquitylation and p97-mediated helicase unloading [510], [511], [512]. When CMG remains within a typical fork structure, for instance at ICLs, unloading cannot occur because CMG still engages the lagging strand template. Rather, CMG removal is triggered via TRAIP (TRAF-interacting protein)-mediated ubiquitylation in trans, mediated by the incoming CMG [reviewed in [513]]. What would happen at a structure-arrested CMG largely depends on the resulting architecture of the fork upon convergence. The most likely outcome is disengagement of the lagging strand template from the stalled CMG, as this strand serves as the tracking strand for the converging CMG. In such a scenario both CMGs would unload, similar to canonical termination, and a ssDNA gap would be left on the leading strand, spanning from the secondary structure to the end of the last Okazaki fragment of the converging fork (Fig. 4A, left). In summary, we propose that long-lived CMG arrest leaves toxic ssDNA gaps that might channel into nucleolytic processing and DSB formation (see Box 2 for more detail, and Fig. 4C).

Box 2 **Scenarios beyond S phase.**

**Late S to G2/M:** As cells finish replication, secondary structures and other obstacles at CFS and structure-prone loci can leave late replication intermediates (LRIs) and ssDNA gaps that persist beyond the normal S/G2 completion window [48], [509], [565], [566]. During S/G2, ATR signalling stabilises stalled forks and coordinates repair, while the RecQ helicases WRN and BLM unwind secondary structures to prevent fork collapse at fragile, structure-prone loci [47], [48], [565]. By late G2/M, the SLX4 scaffold assembles the mitotic ‘SMX’ tri-nuclease MUS81-EME1 (Mus81-Mms4), SLX1-SLX4 (Slx1-Slx4) and XPF-ERCC1 (Rad1-Rad10) and the backup resolvase GEN1 gains access after nuclear envelope breakdown to resolve lingering junctions, acting in parallel to, but distinct from, SMX [567], [568], [569], [570], [571]. Checkpoint signalling (ATR-CHK1/WEE1) restrains MUS81-dependent cleavage during S phase but promotes its activation at G2/M, shifting nuclease access to late intermediates [572], [573], [574]. Loss of CHK1/WEE1 or ATR-hyper-CDK (cyclin-dependent kinase) signalling precipitates MUS81-dependent breaks; conversely, in fission yeast Rad3/ATR directly stimulates Mus81-Eme1 (MUS81-EME1) at G2/M [575], [576], [577], [578].

**Early mitosis:** Under-replicated DNA can be resolved either by SMX-triggered MiDAS, which is a BIR-like fill-in, or by continued fork-coupled synthesis that extends from late G2 into early mitosis [579], [580], [581], [582]. Premature or excessive activation of nucleases is pathological as it generates DSBs and mis-segregation [568], [570], [571], [572], [574], [580], [581]. At AT-rich fragile sites, direct evidence shows that the FRA16D Flex1 (AT)_n_ repeat undergoes structure-selective endonuclease cleavage: in budding yeast, Flex1-dependent chromosome breakage requires structure-selective endonucleases [138]. In MSI human cells, expanded (AT)_n_ repeats become substrates for MUS81-EME1 in the absence of WRN, producing repeat-boundary-enriched DSBs and chromosome shattering [65]. This is consistent with models placing MUS81/XPF-ERCC1 at AT-rich CFS cores in late S/G2-mitosis [565]. In BRCA-deficient cells, TRCs and R-loops bias under-replication toward MiDAS [583]. In early mitosis, TOPBP1-CIP2A complexes help tether and protect broken or under-replicated chromatin [584], [585] [reviewed in [586], [587]]. In BRCA-deficient cells, the CIP2A-TOPBP1 complex controls pathway choice: regulation of TOPBP1-SLX4 assembly by CDK1 drives SMX-dependent MiDAS, while CIP2A also promotes Polθ-MMEJ, dual dependencies that underlie synthetic lethality when CIP2A is lost [588].

**Late mitosis and next S phase:** Unreplicated regions manifest as ultrafine DNA bridges (UFBs) and chromatin bridges in anaphase, which can trigger genome instability (e.g micronuclei, DNA damage) if not properly resolved [589], [590]. Their resolution is actively regulated yet intrinsically break-inducing: actomyosin constriction promotes initial failure, and the midbody endonuclease ANKLE1 (LEM-3 in *Caenorhabditis elegans*) nicks/cleaves bridges near abscission [591], [592]. These regulated routes reduce lethal mechanical rupture yet still fragment chromosomes, promoting micronuclei, chromothripsis, and cGAS-STING activation [593], [594], [595], [596]. Recent work shows that micronuclei frequently accumulate persistent G4 DNA, linking G4-mediated, Pol η/PrimPol-dependent replication stress to micronucleus-associated genome instability [132]. Strand discontinuities that escape repair provide a strand-biased route to breakage on fork encounter in the subsequent S phase: a leading strand nick causes CMG run-off and a single-ended DSB, whereas lagging strand nicks can yield single- or double-ended breaks depending on bypass and convergence [510], [597], [598].

Interestingly, during ICL repair, unhooking of the ICL after CMG unloading by incisions made by SSEs such as XPF-ERCC1 generate DSBs [514], [515], [516], [517]; SSEs are potentially recruited to such stalled forks via the Fanconi Anemia pathway [reviewed in [513]]. Importantly, a recent single-molecule study has shown that the FANCD2-FANCI (Fanconi anemia complementation group D2 and I proteins) complex recognises ss-dsDNA junctions at stalled forks generated by DNA obstacles [518]. By analogy, CMG arrested at leading strand structures could potentially backtrack to allow FANCD2-FANCI binding, leading to a nuclease-mediated DSB. Although it is tempting to directly compare structure-induced CMG arrest with ICLs, the interpretation should be considered with caution. ICLs are static, covalent crosslinks that tether the two strands, whereas secondary structures are dynamic, non-covalent folds. Furthermore, a stable structure could be wedged within the central channel of CMG [466], whereas ICLs lie ahead of CMG. Overall, while incision at secondary structures is plausible by analogy to ICLs, it remains untested.

Another possible outcome is fork reversal, which can similarly lead to formation of ssDNA gaps (see Model 2 A). Although fork reversal is generally thought to be triggered by fork uncoupling, some studies show that CMG stalling by torsional stress and ICLs can trigger reversal [517], [519], [520]. More recent work shows that Rad51 can drive fork reversal while maintaining CMG at the fork [521].

#### 2.3.2 Model 2: fork uncoupling

As described in Section 2.1, a secondary structure on the leading strand will first be encountered by CMG. This can lead to transient fork pausing and CMG bypass. In this scenario, Pol ε will encounter the structure, causing fork uncoupling and exposure of ssDNA. Alternatively, de novo structures could form between CMG and Pol ε, resulting in the same outcome of fork uncoupling. There are two major tolerance mechanisms that can occur upon fork uncoupling: reversal or repriming. The RPA-ssDNA platform recruits factors that can promote either outcome. RPA directly tethers the translocase SMARCAL1 (SWI/SNF-related, matrix-associated, actin-dependent regulator of chromatin, subfamily A-like 1) to promote fork reversal [522], [523] and supports BRCA2-mediated loading of RAD51 to drive reversal and protect the regressed arms [521], [524]. Alternatively, RPA can recruit PrimPol through its RPA-binding motifs, stimulating its primase activity [428], [525], [526].

##### Model 2A: fork reversal

Fork reversal is a protective mechanism that can be triggered by fork pausing and uncoupling induced by replication stress factors such as HU [524], APH [527], and ICLs [517]. Although direct evidence is lacking, there is correlative evidence suggesting that secondary structures can trigger fork reversal. At Friedreich's ataxia-associated (GAA)_n_ triplex-forming repeats, 2D gels and EM studies reveal fork pausing with frequent reversal [86], [528], [529]. However, these studies cannot determine whether fork reversal is directly triggered by triplex structures. At the AT-rich Flex1 element within the CFS FRA16D, which can extrude hairpins or cruciforms, FANCM and BLM independently suppress DSBs and fragility under replication stress [281], [530], [531]. This provides indirect evidence that fork reversal may occur, given both FANCM and BLM’s fork reversal activities [532], [533], [534], [535]. Lastly, the DNA translocase HLTF (Helicase-like transcription factor), which can catalyse fork reversal in vitro [536], [537], has also been proposed to unwind G4s [538] and triplexes [529]. HLTF can also polyubiquitinate PCNA [539], which could possibly recruit ZRANB3 [540]. Therefore, HLTF-mediated resolution of G4s could also promote fork reversal, either via HLTF’s own reversal activity and/or via PCNA ubiquitylation-mediated recruitment of ZRANB3.

Reversed forks can restart once the obstacle has been cleared, after being properly processed by repair and remodelling enzymes [reviewed in [541], [542]]. However, reversed forks can potentially be detrimental since the reannealed nascent strands resemble single-ended DSBs (seDSBs) prone to nucleolytic degradation. Reversed forks are protected by multiple mechanisms that involve RAD51, BRCA1 (Breast cancer type 1 susceptibility protein), and BRCA2 [reviewed in [542], [543]]. However, protection can fail in BRCA1/2 mutants [544] or due to exhaustion of protection factors. Furthermore, the restart of reversed forks requires the regulated and controlled action of exonucleases: MRE11 (Double-strand break repair protein MRE11), DNA2 and EXO1 (Exonuclease 1), and MUS81 (structure-specific endonuclease subunit MUS81). Therefore, mis-regulation of these nucleases would lead to uncontrolled degradation of reversed fork intermediates, leading to formation of ssDNA gaps (Fig. 4A, centre). Furthermore, these ssDNA gaps could yield DSBs within the same S phase; uncontrolled degradation of the nascent strand would form a 3’ flap that is a substrate for MUS81 cleavage on the template strand, causing a DSB [543].

##### Model 2B. PrimPol repriming

At uncoupled forks, PrimPol can reprime downstream, creating a post-replicative gap [428], [430]. Repriming has been observed at secondary structures: in the promotor-proximal G4 at the BU-1 locus in chicken DT40 cells, PrimPol binds G4s for repriming, preserving transcription state across the locus [123], [124], [430]. Other studies in DT40 cells also show that PrimPol-dependent repriming occurs at secondary structure forming sequences such as G4s and triplexes [431]. Structure-uncoupled forks may behave similarly to DPC-uncoupled forks, with both undergoing repriming. DPCs are either degraded, enabling CMG traversal, or are skipped over in an RTEL1-dependent manner. Both events create downstream ssDNA compatible with repriming [470], [489]. This paints a general picture where, regardless of the insult, uncoupled forks drive PrimPol activity. Consistent with this, PrimPol-dependent repriming has also been observed after UV irradiation [545] and during interstrand-crosslink traversal [526].

Repriming by PrimPol can be detrimental since it leaves a vulnerable post-replicative ssDNA gap (Fig. 4A, centre). This gap can persist in conditions such as low RPA [495], [546], frequent repriming, and resection by nucleases. To counter this, gaps can be filled by TLS polymerases, an error prone solution leading to mutations and genomic instability [430], [442], [547], [548], [549], [550]. Pol ζ can traverse hairpins and suppress deletions, making TLS engagement decisive for whether gaps are resolved at this point [422], [442].

Notably, recent polymerase-usage maps now capture strand- and timing-dependent TLS deployment; integrating these with strand-specific DSB and replication mapping could help establish the relationship between TLS-mediated gap filling and fork-proximal breakage at structure-rich loci [551], [552].

Overall, in both structure-induced uncoupling outcomes, reversal or repriming, we consider it likely that ssDNA gaps are generated within the same S phase (Fig. 4A, B). Such ssDNA gaps are inherently vulnerable to cleavage (see Section 2.4) and can be converted into DSBs later during the cell cycle or in the next S phase (Box 2 and Fig. 4C).

#### 2.3.3 Model 3: lagging strand defects

##### Model 3A. Template strand gaps

Pre-formed structures on the lagging strand template are unlikely to block CMG. Rather, pre-formed or de novo structures are most likely to impact Pol δ (Fig. 4A, right). For instance, local R-loops that expose a G-rich lagging strand template promote G4 folding and lead to Pol δ inhibition [465]. Indeed, lagging strand G4s impede replisome progression in *Saccharomyces cerevisiae* in live cell imaging fluorescent assays [129]. However, Pol δ has inherent strand displacement activity, which is likely sufficient to promote replication through hairpins and weak G4s; therefore, only stable structures pose a threat [287], [295]. Furthermore, one study found that CFS-stalled Pol δ dissociates from the template, likely due to an inability to replicate through a structure [553]. The discontinuous nature of lagging strand synthesis means that disrupted synthesis of one Okazaki fragment will not affect subsequent fragments. Pol δ will resume replication of the nascent strand from the next primer along, essentially skipping over the impediment, which also occurs with small lagging strand lesions [473], [474].

The result is a small nascent strand gap between the stall site and the 5’ end of the previous Okazaki fragment [554]. The size of this gap will be dictated by the last priming event, and will typically be up to the size of an Okazaki fragment [465]. Some cellular studies suggest that lagging strand G4s induce DSBs, potentially in a MUS81-dependent manner [555], [556]; however, the underlying mechanism is unclear.

##### Model 3B. OFM impairment

In addition to forming on the template lagging strand, structures could also form on the nascent lagging strand during strand displacement synthesis. Specifically, long 5′ flaps could fold de novo into hairpins, triplexes or G4s. Such structures are poor substrates for FEN1, since they conceal the 5’ end of the flap required by FEN1 for cleavage (Fig. 4A, right) [491], [557], [558] and often require DNA2 and/or BLM [370], [559], [560], [561].

Overall, structures on the nascent lagging strand impair OFM, leaving unligated nicks or short ssDNA gaps, which can drive repeat expansions/contractions [562]. Paradoxically, while PIF1 is required for replication through lagging strand G4s [129], [287], [465], it also enhances Pol δ strand displacement [563], [564], which could contribute to flap formation and hence OFM-blocking structures. How those two opposing activities are regulated is unclear. Lastly, impairment of OFM by secondary structures could expose more RPA-ssDNA, and in turn, potentially trigger fork reversal, which can also lead to formation of ssDNA gaps (see Model 2 A).

### 2.4 Added layers of complexity

Since cells are complex environments, we account for additional factors that can exacerbate structure-induced gaps and breaks during the same S phase, as described below:

#### 2.4.1 Gaps or breaks?

Overall, our models all suggest that replication of secondary structures results mainly in ssDNA gaps. We consider it unlikely that breaks form in the same S phase, except in the case of nuclease-mediated cleavage. However, these gaps can be converted into breaks later in the cell cycle, in G2 and mitosis, or even in the next cell cycle (Box 2). Consistent with this, in vivo work at G-quadruplex loci shows that failure to replicate across a single G4 can generate a heritable ssDNA gap that is converted into a DSB in subsequent cell cycles, providing a concrete example of the type of gap-to-break pathway we consider here [131]. This leads us to a more general question: which outcome is more toxic - gaps or breaks? Traditionally, breaks have been viewed as the most cytotoxic but recent evidence suggests that ssDNA gaps may be intrinsically toxic, not merely as precursors to breaks [599], [600]. One potential mechanism is that ssDNA gaps can sequester various limiting factors such as RAD51, which can over-accumulate on excess ssDNA and inhibit its other activities [601]. Similarly, persistent gaps may titrate out free RPA, causing RPA exhaustion and fork catastrophe [546], [602]. Moreover, PCNA might be retained at junctions, especially on the lagging strand, which would restrict its availability for active forks [507], [508].

#### 2.4.2 Multiple obstacles and synergy between structures

Because C-rich strands can form iMotifs and their complementary G-rich strands can form G4s, many loci can, in principle, host either structure on either strand; in model duplexes these structures are largely mutually exclusive [112], [145], [147], [603], [604]. Similarly, transcription-generated R-loops expose the displaced DNA strand; when that strand is G-rich, it can fold into a G4, producing G-loops [118], [176], [605], [606]. Therefore, when both strands can form structures, the odds that at least one becomes structured increases. Conversely, certain sequences can form multiple or competing structures on the same strand. Long C-rich repeats can adopt both hairpin and iMotif conformations, and recent work shows that flanking inverted repeats that form a stem-loop around the iMotif further accelerate its folding and increase its stability [152]. In such contexts, structure-resolving proteins must act against this additional stabilisation. For example, within single C-rich repeats on the same template, the efficiency of PCBP1 (Poly(rC)-binding protein 1) in iMotif unwinding is reduced when a hairpin is present, thereby increasing the chance that an iMotif persists into S phase. Accordingly, reducing hairpin propensity alleviates fork pausing [607].

#### 2.4.3 ssDNA vulnerability

Long stretches of ssDNA are more prone to physical breakage than dsDNA [608]. Notably, upon replication, iMotifs can generate DNA breaks in vitro [295]. Whether this reflects enzymatic activity by a replisome factor or an inherent weakness in the structure, remains unresolved. On replication-exposed ssDNA, APOBEC3A/B (Apolipoprotein B mRNA editing enzyme catalytic subunit 3 A/3B) preferentially deaminates cytosines in hairpins; these sites are then processed by UNG (Uracil DNA glycosylase) and APE1 (DNA-apurinic/apyrimidinic Endonuclease 1) into AP (abasic) sites [47], [609], [610] (Fig. 4B, left). By analogy, C-rich iMotifs could perhaps increase APOBEC-accessible ssDNA when unfolded, but direct evidence is lacking. Because RPA normally coats ssDNA, APOBEC access increases where RPA is limiting or displaced, which aligns with APOBEC3A/B hairpin-targeting in gaps or flaps [611]. Additionally, oxidized and AP sites can undergo spontaneous scission, with nucleosomes accelerating AP-site cleavage [608], [612], [613], [614]. In fact, when such gaps are present near secondary structures, they can likely become APE1-bound during base excision repair (BER) [615]. AP sites embedded in G-rich DNA can promote G4 folding (e.g. at the KRAS promoter) and its cleavage is influenced by topology: AP sites in certain G4 folds (especially telomeric) are poor substrates, favouring either persistent APE1-bound obstacles or inefficient incision to gaps [615], [616], [617], [618]. Consistent with intrinsic AP-site fragility, reconstituted budding yeast replisomes encountering AP site templates yield more ‘broken-fork’ products than with CPDs [473].

#### 2.4.4 Cleavage by nucleases

It is conceivable that in addition to cleaving stalled fork structures, nucleases might directly target secondary structures (Fig. 4B, right). For example, DNA2 helicase/nuclease cleaves G4s in vivo, which is stimulated by the mismatch repair protein MSH2 (MutS homolog 2) [619]. Also, genetically encoded lagging strand structure-forming repeats show MUS81-dependent, replication-coupled breaks [555], [556]. Furthermore, expanded cruciform-forming (AT)_n_ repeats in MSI cancers are unwound by WRN; in the absence of WRN, these become substrates for MUS81-EME1/SLX1-SLX4 cleavage [65], [379]. The MMR complex MutLγ (MLH1-MLH3 heterodimer) is an endonuclease that has also been shown to nick DNA opposite to DNA loops formed at disease-causing trinucleotide repeat sequences [620] (Fig. 4B, left).

#### 2.4.5 Topoisomerases as protein-bound nicks

Topoisomerase binding can influence structures and their propensity to generate breaks. TOP1 (Topoisomerase 1) can become trapped at G4s, creating protein-bound nicks [621] [reviewed in [622]] (Fig. 4B, left), which have been shown to impede replication [623]. Indeed, cleavage-defective Top1 mutants trapped at G4 motifs sharply increase G4-associated genomic instability in yeast, highlighting the deleterious consequences of persistent Top1-G4 complexes [624]. Genetic and mechanistic studies identify TOP2A (Topoisomerase 2α) as a major effector of DNA breaks induced by the G4-stabilising ligands PDS and CX-5461, preferentially at the rDNA promotor [625], [626]. Consistent with this, G4 stabilisation in B lymphocytes induces DNA breaks and chromosomal rearrangements at pericentromeric satellite repeats and ribosomal DNA arrays [627]. Moreover, PDS induces DNA double-strand breaks and shows selective toxicity in ARID1A-deficient cells, where defective repair of topoisomerase-induced breaks underpins hypersensitivity [628].

#### 2.4.6 Sensitivity to PARP1 inhibition: gaps or breaks?

PARP1 can sense and control the lifetime of gaps arising near structures. In BRCA-mutant cells, PARP inhibitors induce synthetic lethality. This toxicity is conventionally thought to be due to PARP ‘trapping’ on DNA, leading to formation of DSBs [629], [630] [reviewed in [631], [632]], with current work refining trapping as a kinetic/allosteric retention phenomenon [633], [634]. However, recent opposing evidence instead points to persistent post-replicative gaps as the major toxic trigger, particularly in BRCA1-deficient cells in which excessive resection and/or TLS failure prevent timely gap filling [304], [562], [599], [600], [635]. These ideas align with our models of resultant gaps at structure-stalled forks, reinforcing the outstanding question of why gaps are toxic, and with work showing that unrepaired BER intermediates in template DNA ahead of replication forks trigger fork collapse and sensitise cells to PARP inhibition [636]. A further layer of complexity comes from PARP1’s role in a backup OFM pathway, sensing and processing unligated Okazaki fragments, especially when FEN1/LIG1 are limiting [637]. Accordingly, post-replicative nicks/gaps accumulate upon PARP1 inhibition or FEN1 loss [635]. PARP1 has also been shown to bind G4s, with some G4s directly activating its enzymatic activity and modulating its DNA retention [638], [639]. Finally, APOBEC3A can sensitise to ATR/PARP inhibition through PrimPol-dependent generation of long ssDNA gaps, further increasing cleavage opportunities at structure-associated lesions [640].

#### 2.4.7 Proximity to an origin

Work from the Leffak lab has shown that replication origin position, origin firing characteristics, and repeat tract orientation impact the replication of secondary structure-forming sequences, leading to their instability [555], [556], [641], [642]. These studies utilised artificial constructs containing repeat tracts positioned next to an ectopic early firing c-myc origin at a defined locus in HeLa cells [643], [644]. The results must be interpreted with caution, as both origin placement and integration site may influence instability, and some outcomes may reflect interference with origin firing rather than fork progression. Nonetheless, the results suggest that the local replication programme (origin proximity, timing and fork direction) is an important contextual parameter when considering structure-induce instability.

#### 2.4.8 Accessory helicase trapping/binding

Although speculative, accessory helicases engaged at DNA structures could adopt long-lived, low-turnover DNA-bound states. When persistent, these non-covalent protein-DNA roadblocks may functionally mimic DPCs. Consistent with this, yeast Pif1 binds parallel G4 DNA with high affinity and unfolds it slowly, giving rise to long-lived Pif1-G4 complexes [292], [645], [646] and an ATPase-defective FANCJ mutant acts in a dominant-negative fashion by remaining DNA-bound [647], [648]. This raises the possibility that persistent helicase-DNA complexes convert a potential solution to structure-induced stalling into a new protein roadblock for subsequent forks.

#### 2.4.9 Factors with multiple roles

Many of the factors discussed in this review play various roles at the replication fork and beyond. This makes it much harder to draw concrete conclusions, especially in knock-out studies where all functions are absent. A classic example is BRCA1, which is a central player in multiple steps of HR, but is also important for fork protection. Importantly, a separation-of-function mutant of BRCA1 allows these activities to be disentangled [649].

In OFM, BLM and WRN, together with RECQ1, FEN1 and DNA2, cooperate to process structured or long flaps, while PIF1 and FANCJ handle G-rich folds and structure flaps to promote Okazaki fragment completion and limit persistence of unligated intermediates [128], [278], [369], [370], [387], [399], [650]. During fork reversal, RPA-ssDNA helps recruit and activate BLM and WRN, which promote fork regression, processing of reversed forks and protection from nucleolytic degradation [270], [273], [534], [535]. HLTF has been implicated in both reversing structure-stalled forks and G4 unwinding, potentially bridging reversal capacity with direct structure resolution [536], [538]. In DNA repair, BLM plays significant roles in HR resolving double HJs, in DNA end resection, and in the MMR pathway [651], [652], [653], [654]. WRN is involved in HR, non-homologous end-joining (NHEJ) and BER [655], [656], [657], and its exonuclease and helicase activities have separate roles at reversed forks [658]. FANCJ is a key player in the Fanconi Anemia repair pathway, with roles in ICL repair, HR and TLS-mediated fill-in [339], [659], [660]. Because these factors may already be engaged at stalled forks or during their repair, they are pre-positioned for opportunistic structure resolution; conversely, their presence at structures may affect their ability to carry out their other roles

Additionally, factors central to break-induced replication (BIR) also have multiple roles. PIF1 acts as a helicase during BIR but also unwinds secondary structures, with its structure-resolving activity being independent of PCNA or Pol δ [223], [286], [288], [564], [661]. The non-essential Pol32/POLD3 subunit of Pol δ is required for BIR function and is also shared with TLS pol ζ, complicating interpretation of Pol δ/Pol ζ defects. [662], [663], [664], [665], [666]. PCNA likewise promotes BIR-associated synthesis and template switching and serves as a central interaction hub for many replication and repair proteins, with most interactors competing for the same binding interface [564], [661], [667], [668].

Due to their multiple roles, it is important when studying these proteins and pathways to uncouple functions as much as possible. A combination of defined biochemistry, allowing precise control of specific components, with separation-of-function mutants, should aid in separating their roles in DNA secondary structure resolution from their other roles.

### 2.5 Repair and (epi)genome instability

While we focused on how structures cause replication-dependent gaps and breaks, cells must repair the damage to preserve genome integrity [reviewed in [669], [670], for G4-specific aspects in repair refer to [671]]. Much of what we know about broken fork repair comes from engineered, protein-mediated fork obstacles that behave as hard CMG stalls. In human cells, Tus/Ter arrays generate both seDSBs and convergent double-ended DSBs (deDSB) and have been used to define the balance between BIR-like restart and HR-mediated repair [483], [672]. In *Schizosaccharomyces pombe*, the polar RTS1/Rtf1 barrier likewise imposes controlled fork arrest and Rad51-dependent recombination, with Pol δ/POLD3 and Pfh1 (PIF1) contributing to BIR-like synthesis and long-tract copying [415], [673], [674], [675]. These systems do not recapitulate the dynamics of secondary-structure folding per se, but they provide a mechanistic insight for processing of replication-coupled DSBs. Tus/Ter and RTS1/Rtf1 are best viewed as ICL-like in geometry as they hard-stall CMG, but not in chemistry: ICLs tether the two strands whereas protein obstacles are non-covalent roadblocks.

Interestingly, proteomics at broken forks indicates an ATM-directed program that promotes resection while dampening the canonical RNF168/BRCA1 ubiquitylation cascade, consistent with nicking studies showing that at replication-coupled DSBs the initial end resection is BRCA1-independent, distinguishing these from canonical breaks [597], [676], [677]. seDSBs from single-fork collapse lack a second end and bias towards BIR-like restart, whereas SMX-triggered mitotic DNA synthesis (MiDAS) provides a mitotic fallback at under-replicated sites [579], [580], [581], [678]. deDSBs formed at convergence are repaired predominantly by BRCA1/RAD51-dependent HR, with resolution by MUS81-SLX4/GEN1 SSEs in late S/G2 [597], [679], [680], [681], [682]. These routes are error-prone, yielding long-tract copying, tandem duplications, and complex structural variants [683], [684]. NHEJ is largely dispensable for replication-dependent DSBs in yeast and human systems, whereas Pol θ-mediated end-joining (MMEJ/TMEJ) can patch isolated ends/gaps with microhomology-linked indels [679], [685]. In line with this, persistent G-quadruplex barriers in *Caenorhabditis elegans* produce both small, gap-sized deletions and more complex rearrangements at G4 motifs via Polθ-mediated end joining [131]. At expandable repeats, MMR (MutSβ/MutLγ) engagement of looped intermediates and BIR favour large one-step expansions [418], [620], [686], [687], [688]. Fork convergence suppresses BIR and promotes HR in fission yeast, though HR can complete without overt convergence in some human contexts [678], [679]. Fork-processing also modulates the route of repair. DNA2 suppresses HR-restarted replication and checkpoint activation at stalled forks, promoting normal completion and preventing proliferation arrest [689], [690].

#### 2.5.1 Epigenetic consequences

In addition to affecting the stability of DNA, secondary structures can also perturb epigenetic inheritance. For instance, G4s prolong fork uncoupling, which decouples DNA synthesis from parental histone recycling, and thereby interferes with timely RCNA [123], [124]. In fact, any uncoupling scenario is likely to interfere with nucleosome inheritance, as ssDNA is a poor substrate for nucleosomes [691]. Limiting TLS across G4s can further erode epigenetic marks and alter gene expression [123]. Stabilising G4s by dNTP depletion exacerbates these epigenetic defects [125]. Structure-induced epigenetic disruption may affect chromatin state, since stalled forks are shown to promote heterochromatinization [692] [reviewed in [693]], and FANCJ is shown to constrain heterochromatin spreading at G4s [261]. Additionally, replication stress (e.g., HU) produces excess ssDNA and perturbs histone recycling, affecting H3K9 methylation and local heterochromatin [694]. Repair pathway choice can be influenced by epigenetic marks [680], [695], [696], [697], [698], [699], [700], so structure-driven epigenetic changes can impact their own repair. Nuclear positioning also shapes repair pathway choice at secondary-structure loci. Relocation to nuclear pores was found to stabilise CAG/CTG hairpin-forming repeats and channel repair towards less deleterious routes [701], [702] [reviewed in [703]]. In summary, epigenetics can influence the repair outcomes of structure-induced instability, but secondary structures can impact RCNA-mediated epigenetic inheritance, thereby fuelling a potential (epi)genomic instability cycle.

### 2.6 ‘Gaps’ in the field and future challenges

This review has summarised how DNA secondary structures can challenge replication fork progression and integrity when unresolved. Although genome instability at these loci is well documented, the mechanistic paths from fork encounter to downstream instability remains underdefined [48], [509]. Here, our proposed framework integrates strand context and fold timing.

Testing the validity of these models presents several technical, conceptual, and integrative challenges. Technically, we lack reliable and quantifiable in vivo detection methods for secondary structures that are, crucially, coupled to fork movement [methods reviewed in [43], [113]]. The same is true for following the fate of gaps and nicks, whether they mature into breaks inside cells or not. Many experimental set-ups exist, but none demonstrate how a structure-induced stall progresses mechanistically to gaps or breaks. Rather, existing models are inferred from static genetic outcomes and correlations. Widely used nickase and overexpression systems help map possibilities but may not reflect the native landscape [704]. The use of replication stress inducing agents (e.g., hydroxyurea and aphidicolin) produces genome-wide, random, and heterogenous stalling on both strands, while preventing any possibility for recovery or repair, all while inducing a global checkpoint response. While this might be useful for producing strong and reproducible readouts, it is unclear how well this reflects individual events under normal conditions. Strand specificity is another sticking point: in cells, it remains hard to assign outcomes to the leading versus lagging strand. Single-molecule localisation microscopy has offered valuable snapshots, yet its strand assignment and mechanistic resolution are limited [111], [705]. Methodologically, we require readouts that can measure uncoupling in real time with minimal perturbation, ideally able to distinguish between downstream intermediates (reversal, repriming, persistent nicks), and then track which of these routes culminate in gaps and/or breaks. Gaps might be difficult to discriminate from other ssDNA containing intermediates, such as resected overhangs [706], [707]. In parallel, chromatin-capture and live-cell imaging approaches already provide partial readouts of replisome-proximal RPA/PCNA and fork components [708], [709], [710], [711], and single-molecule CMG imaging illustrates how helicase fate (retention, bypass and unloading) can be tracked directly [712]. Scaling such methods to higher temporal resolution and coverage will be essential to generate quantitative maps of CMG fate and RPA/PCNA retention and post-translational modifications and thereby define the windows for reversal versus repriming and late-S/G2 nuclease access. In the near future, allele- and strand-resolved single-molecule/long-read approaches should become standard to phase gaps, nicks, and rearrangements to the same allele/strand, rather than averaging across cells [713].

Conceptually, break formation is likely to be complex, and it might not be possible to assign a distinct break mechanism to each stall type. The same motif on a given strand may generate a variety of intermediates depending on local context, making causal inference in vivo especially challenging. Many factors contribute to DSB formation and repair, so cataloguing proteins (e.g., by proteomics) tells us which proteins are present, but not what they do [677], [709], [714], [715]. Given recent advances in reconstitution approaches, a productive path is to build from reductionist biochemistry, in which the system is highly defined (yet simplified), then move back into cells with informed, acute perturbations, separation-of-function alleles and new in vivo methodologies. Finally, addressing these questions requires collaboration between multiple fields to allow analysis of strand-specific replication of structured DNA, fork stalling and uncoupling, and mechanisms of DSB formation and repair. Each community brings powerful techniques, and progress will require a wider, explicitly cross-disciplinary approach to follow the same locus from biochemistry to single-molecule imaging to cell-based genomics. This should help draw true mechanistic links.

Understanding the fundamental mechanisms by which structure-induced stalls produce breaks has important clinical implications. This is exemplified in MSI tumours, where WRN counteracts structure-born fork obstacles; stabilising folds or prolonging daughter-strand gaps should heighten WRN reliance [65], [716], [717]. In HR-defective settings, G4 ligands tend to channel under-replicated regions into RAD52/POLD3-dependent MiDAS/BIR-like completion, suggesting synthetic-lethal combinations with RAD52/POLD3 or PIF1 perturbation [580], [581], [661]. PARP inhibition shifts obstacles toward gaps/nicks, predicting MUS81-dependent termination cleavage and enhanced sensitivity to G4 stabilizers in HR-defective contexts [520], [635], [718], [719]. Consistently, recent work implicates RAD51 and other DNA-damage signalling factors in tuning PARP inhibitor responses in BRCA-null cancers [601]. In addition, emerging data in BRCA2-deficient cancers reveal G4-enriched structural variant breakpoints, replication slowing with G4 stabilisation, and PIF1 overexpression/dependence [141].

Taken together, we suggest that most structure-induced obstacles first manifest as strand-specific, ssDNA gaps rather than immediate breaks. Leading strand encounters bias toward reversal or PrimPol-dependent repriming, whereas lagging-strand structures chiefly perturb OFM. Progress now hinges on strand-specific, time-resolved measurements that connect CMG fate, uncoupling length, and gap processing to mutational outcomes at single loci. Aligning reductionist biochemistry with live-cell and genomics readouts should move the field from correlation to mechanism.

## CRediT authorship contribution statement

**Billie Delpino:** Writing – review & editing, Writing – original draft, Visualization, Data curation, Conceptualization. **María Fernández-Casañas:** Writing – review & editing, Writing – original draft, Visualization, Data curation, Conceptualization. **Aditya Sethi:** Writing – review & editing, Writing – original draft, Visualization, Data curation, Conceptualization. **Gideon Coster:** Writing – review & editing, Writing – original draft, Supervision, Funding acquisition, Conceptualization.

## Declaration of Competing Interest

The authors declare that they have no known competing financial interests or personal relationships that could have appeared to influence the work reported in this paper.
